# Coupled discrete phase model and Eulerian wall film model for numerical simulation of respiratory droplet generation during coughing

**DOI:** 10.1038/s41598-022-18788-3

**Published:** 2022-09-01

**Authors:** Hitomi Anzai, Yugo Shindo, Yutaro Kohata, Masahiro Hasegawa, Hidemasa Takana, Tetsuro Matsunaga, Takaaki Akaike, Makoto Ohta

**Affiliations:** 1 Institute of Fluid Science, Tohoku University, Katahira 2-1-1, Aoba-ku, Sendai, Miyagi 980-8577 Japan; 2 Graduate School of Engineering, Tohoku University, Aoba 6-3 Aramaki, Aoba, Sendai, Miyagi 980-8578 Japan; 3 Present Address: Graduate School of Biomedical Engineering, Tohoku University, 6-6 Aramaki-Aza-Aoba, Aoba-ku, Sendai, Miyagi 980-8579 Japan; 4 Department of Environmental Medicine and Molecular Toxicology, Tohoku University Graduate School of Medicine, 2-1 Seiryo-machi, Aoba-ku, Sendai, Miyagi 980-8575 Japan

**Keywords:** Diseases, Mathematics and computing

## Abstract

Computational fluid dynamics is widely used to simulate droplet-spreading behavior due to respiratory events. However, droplet generation inside the body, such as the number, mass, and particle size distribution, has not been quantitatively analyzed. The aim of this study was to identify quantitative characteristics of droplet generation during coughing. Airflow simulations were performed by coupling the discrete phase model and Eulerian wall film model to reproduce shear-induced stripping of airway mucosa. An ideal airway model with symmetric bifurcations was constructed, and the wall domain was covered by a mucous liquid film. The results of the transient airflow simulation indicated that the droplets had a wide particle size distribution of 0.1–400 µm, and smaller droplets were generated in larger numbers. In addition, the total mass and number of droplets generated increased with an increasing airflow. The total mass of the droplets also increased with an increasing mucous viscosity, and the largest number and size of droplets were obtained at a viscosity of 8 mPa s. The simulation methods used in this study can be used to quantify the particle size distribution and maximum particle diameter under various conditions.

## Introduction

Pathogens that cause infectious respiratory diseases such as the severe acute respiratory syndrome coronavirus 2 (SARS-CoV-2) are transmitted via respiratory droplets containing lung fluid. Droplets are produced by the stripping and rupture of fluid in the respiratory system^1^, and they are expelled from the body by exhalation phenomena such as coughing, sneezing, and talking. The released droplets evaporate, condense, or hygroscopically expand under the influence of the ambient temperature and relative humidity to propagate through the air while changing their diameter. The droplets can then adhere directly to the conjunctiva, oral mucosa, and nasal mucosa of susceptible individuals to cause infection.

**Figure 1:**
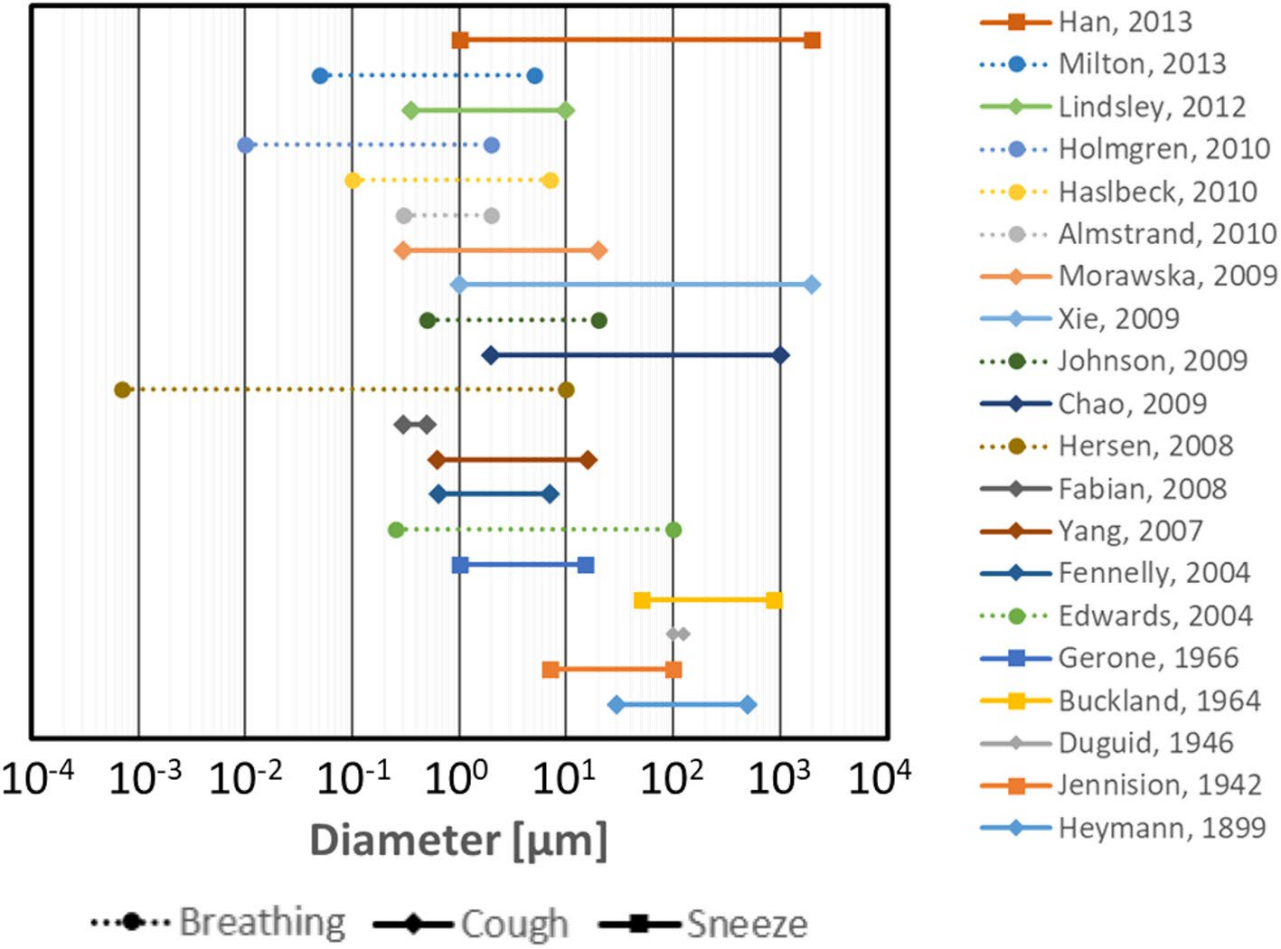
Overview of particle size ranges of respiratory droplets reported in previous studies^8–26^.

Computational fluid dynamics (CFD) is widely used to analyze the spreading phenomena of droplets in the environment^2–7^. For these CFD analyses, the initial conditions for the droplet particles are commonly based on particle size distributions obtained experimentally. Therefore, accurate measurement of the particle size distribution is important. Various measurement techniques have been used, but the particle size distributions reported in the literature vary widely (Fig. 1)^8–26^. Limitations on the resolution and measurement range of various instruments make it difficult to obtain the entire range of the particle size distribution experimentally. Therefore, currently reported results of CFD analyses may only reflect the behavior of a specific particle size. To realize a more accurate analysis of the droplet behavior, a comprehensive particle size distribution needs to be obtained. Numerical simulations may be an effective approach, but simulating droplet formation within the large-scale domain of the respiratory system is a significant challenge.

Previous studies have identified the following mechanisms of droplet formation in the respiratory system: expansion and rupture of bag-like fluid (mucus and saliva) generated in the oral cavity for millimeter droplets, stripping of the airway wall mucosa due to high shear stress, and rupture of the liquid film generated in the cross-section of the bronchi for nanometer droplets^1,27–29^. Although these mechanisms have been studied qualitatively, there are very few examples of quantitative analyses of droplet generation, and there are almost no reports on quantifying the number, mass, and particle size distribution of droplets generated by each mechanism. As a first step to characterize a comprehensive particle size distribution of droplets, this study focused on quantitatively analyzing the stripping mechanism of the airway wall mucosa.

The volume of fluid (VOF) method is commonly used to simulate multiphase flows with a gas–liquid interface. To represent droplet stripping with the VOF method, Pairetti et al.^30^ performed a multiphase flow simulation while using a rectangular wind channel (with a liquid film of 4 × 1 cm^2^) to represent the airway wall mucosa. In their simulation, the air phase (gas) and mucous layer (liquid) were fully resolved on a numerical mesh. Therefore, changes in the gas–liquid interface including droplet formation could be observed in detail. However, tracking microscale and nanoscale droplets in millimeter- to centimeter-scale tubes requires a very fine mesh, which inevitably has a high computational cost.

As an alternative to the VOF method, the Eulerian wall film (EWF) model and discrete phase model (DPM) can be used to simulate droplet generation and its flow in a Lagrangian framework according to Newton’s laws of motion^31^. The EWF model was developed to represent the flow of a thin liquid film based on the phenomenon observed in experimental study^32–35^. The advantage of assuming a thin film is that the liquid film can be expressed as a physical quantity on the surface mesh of a rigid wall, which eliminates the need to resolve the film thickness. The EWF model has been applied to simulating changes in mucosal thickness^36–39^, but these studies focused on changes due to coughing up sputum and did not consider droplets generated during coughing. The EWF model can be applied to simulating droplet generation when coupled with the DPM for mass conservation of the liquid film and for tracking droplet particles in the gas phase. Stripping of the liquid film occurs when the velocity difference between the gas phase and the liquid film on the wall is large. Lopez de Bertodano et al. constructed the experimental system to test the film dryout in annular flow and modelized the droplet generation induced by shear^33^. If the shear is sufficiently large, Kelvin–Helmholtz waves are generated on the surface of the film, which grow and eventually strip the droplet off the film surface^33,34^. And if the crossing angles of the planes are sufficiently large at the edges and the inertia of the liquid film is sufficiently large, the liquid film will separate and become droplets^35^. To the best of our knowledge, there have been no reports on simulating droplet generation and droplet tracking in a 3D airway model.

The objective was to identify quantitative characteristics related to droplet generation during coughing. To generate the droplets induced by airflow on airway mucosa, the DPM and EWF model were coupled for CFD analysis. By introducing this coupled scheme, comprehensive droplet distribution can be obtained regardless of the high computational cost due to the ultra-fine mesh. The effects of the airway mucous viscosity and cough flow rate, which contribute to droplet generation, on the number, mass, and particle size distribution of the droplets were evaluated. The comprehensive distributions obtained in the present study provide the wider scale of droplet generation, from nanometers to millimeters, and can be applied to CFD analysis for droplet spreading in air.

## Methods

### Airway geometry

**Figure 2:**
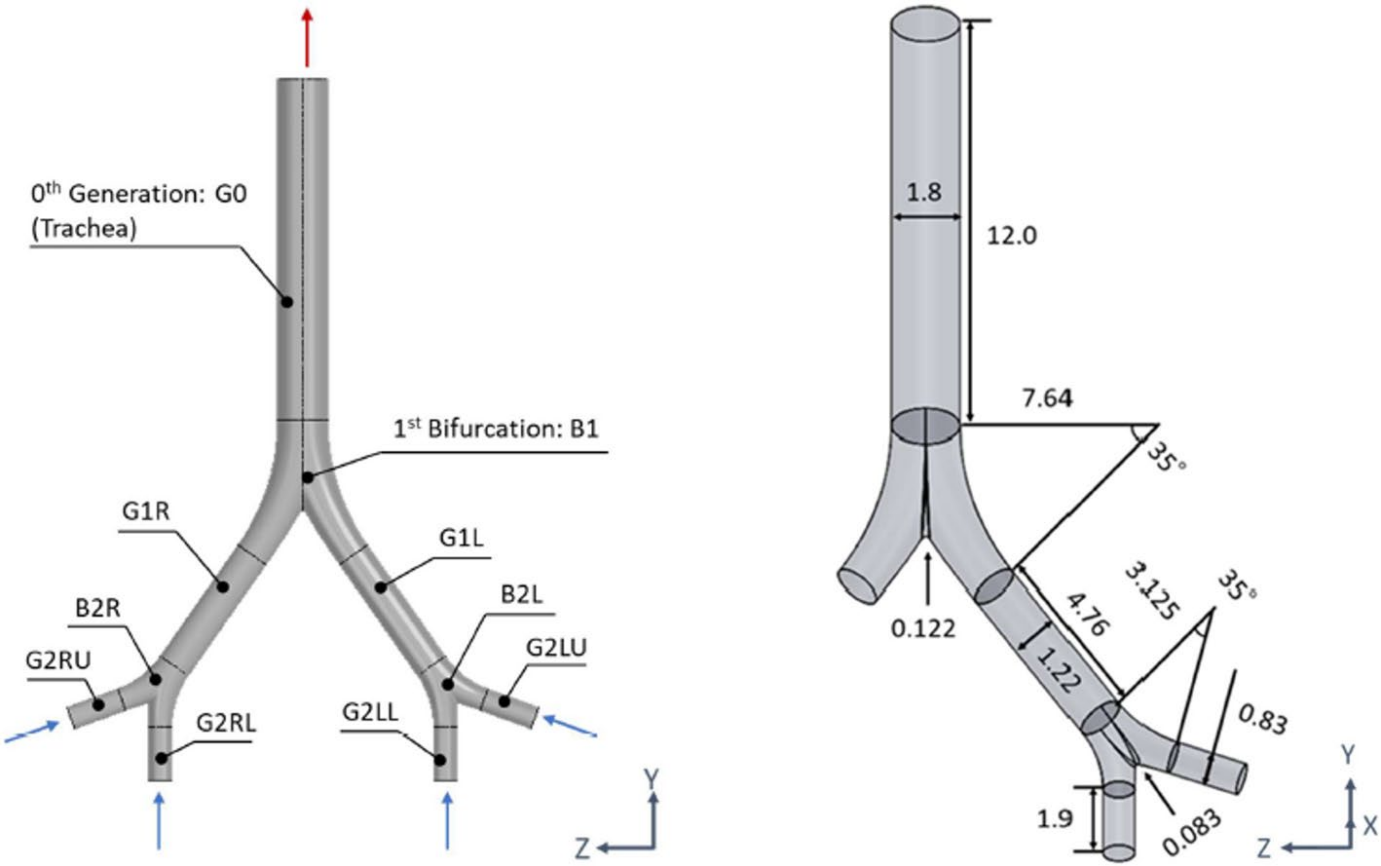
Airway model: (left) name of each part; (right) diameter, length, and angle.

An ideal symmetric lower airway model (Fig. 2) was constructed by using SolidWorks 2019 (Dassault Systems, France) and is based on Weibel’s respiratory system model, which was constructed from his measurements of human lung morphology^40^. The airway model was constructed to represent the airway tree from generation-0 to generation-2 and had five components: the trachea (G0), first branch (B1), first-generation bronchus (G1), second branch (B2), and second-generation bronchus (G2). The left and right sides were designated by L and R, respectively (e.g., G1R represented the first-generation bronchus on the right side). The diameter and length of each bronchus were taken from Weibel’s model. Following Rajendran et al.^41^, the radii of curvature at B1 and B2 were set to 7*d*_1_ and 4.5*d*_2_, respectively, where *d*_1_ was the diameter of G1 and *d*_2_ was the radius of curvature of G2. The lower end of G2 was extended by 30*d*_2_ to represent the entrance region of the airflow. The inlet was at each G2, and the outlet was at G0. All sides of the model were treated as walls of the airway.

### Simulation settings

Unsteady-state calculations of droplet generation were performed by using Ansys Fluent 2020R1 (Ansys Inc., USA) until the peak cough flow point or peak volume time (PVT). The airflow calculated by CFD was used by the EFW model to simulate the behavior of the thin mucous film and generate droplet particles. The generated droplets were then tracked by using the DPM. The coughing airflow was assumed to be an isothermal and incompressible ideal gas with a density and viscosity of 1.185 kg/m^3^ and 1.81 × 10^−5^ Pa, respectively^38^. The governing equations for the airflow were the equation of continuity and the Navier–Stokes equations. The cough flow rate at the inlet boundary condition was taken from Gupta et al.^42^, who measured a cough peak flow rate (CPFR) of 1.6–8.5 L/s for 25 healthy subjects. Thus, four different CPFRs were considered for the airflow simulations: 2, 4, 6, and 8 L/s. Because the airway model had four inlets at G2, the cough flow rate multiplied by a factor of 1/4 was set at the four inlets as a mass flow boundary. The outlet boundary was set as the pressure outlet with a gauge pressure of 0 Pa. The time step for calculating the cough airflow was set to 1.0 × 10^−3^ s. The walls were assumed rigid and no-slip. The SST *k*–ω model was applied to represent the turbulence of the coughing airflow.

For the first time step of the simulation, the airway wall was initially covered by a mucous liquid with a thickness of 30 µm, which is the same thickness used by Paz et al.^36,37^. The surface tension of the mucous film was set to 7.2 × 10^−2^ N/m, which is the same as that of water. The density and viscosity of the mucosa were set to 998.2 kg/m^3^ and 1.00 mPa s, respectively. To investigate the effect of the mucous viscosity on droplet formation, simulations were performed with viscosities of 8.25 and 15.5 mPa s at a fixed CPFR of 4 L/s. The time step for the liquid film calculations was set to 1.0 × 10^−5^ s. The critical shear stress (CSS) was set to 1 Pa. The physical properties of the droplet particles were set to be the same as those of the mucous film in the EWF model. The discrete phase was solved by integrating the force balance on the droplets. For CFD-DPM, only the effect of the airflow on the droplets was considered; other forces such as gravity, Saffman’s lift force, rotational force was not applied. CFD-DPM was coupled in one way, so that the effect of the droplets on the airflow was not considered. Liquid evaporation was neglected. An escape boundary for the dispersed phase was set at the outlet; droplets that reached the end of G0 were terminated. The airway wall was set as a trap boundary for the dispersed phase; droplets that adhered to the wall surface after formation were considered to merge back into the liquid film.

To evaluate the quantitative characteristics of the droplets, the number of generated droplets was calculated every time step. Although the EWF model in Fluent could produce droplets up to 1 nm, only particles greater than 0.1 mm, which corresponds to the particle size of SARS-CoV-2, were counted to generate the histograms.

## Results

### Influence of the flow rate

**Figure 3:**
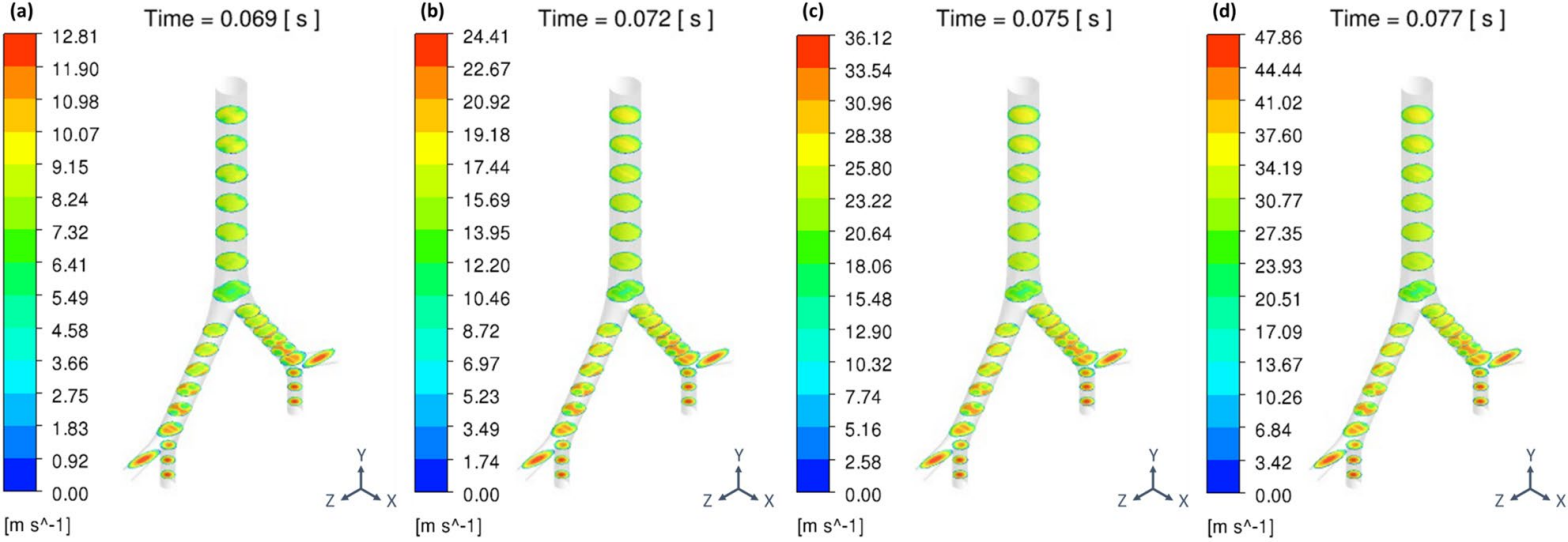
Velocity contours for cross-sections of the airway at PVT.

**Figure 4:**
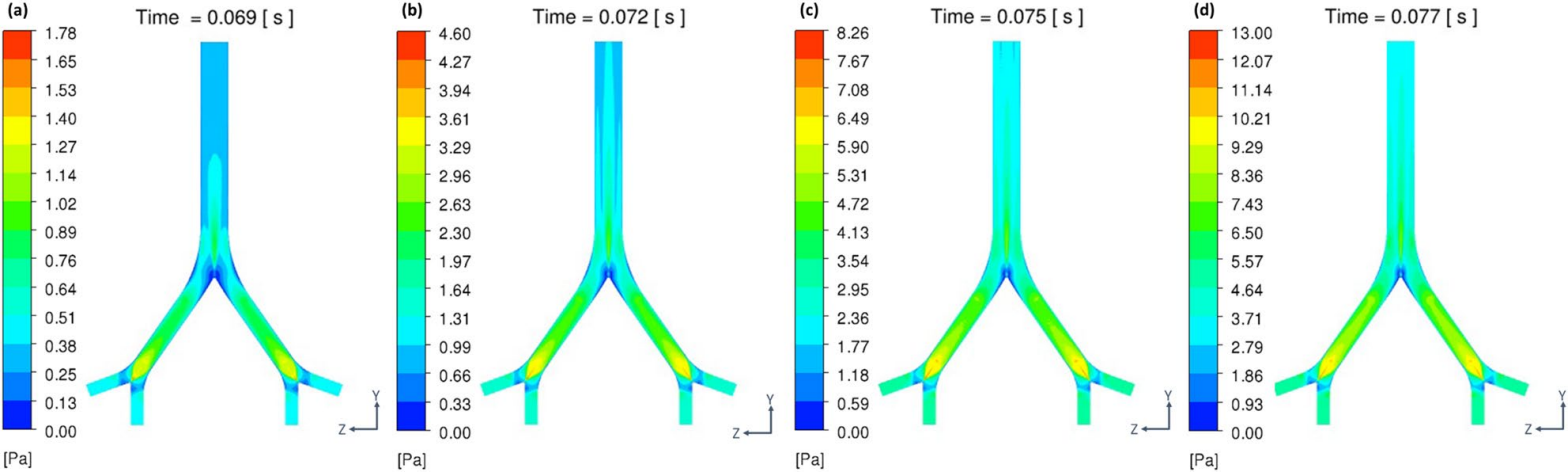
WSS contour on the airway surface at PVT.

**Figure 5:**
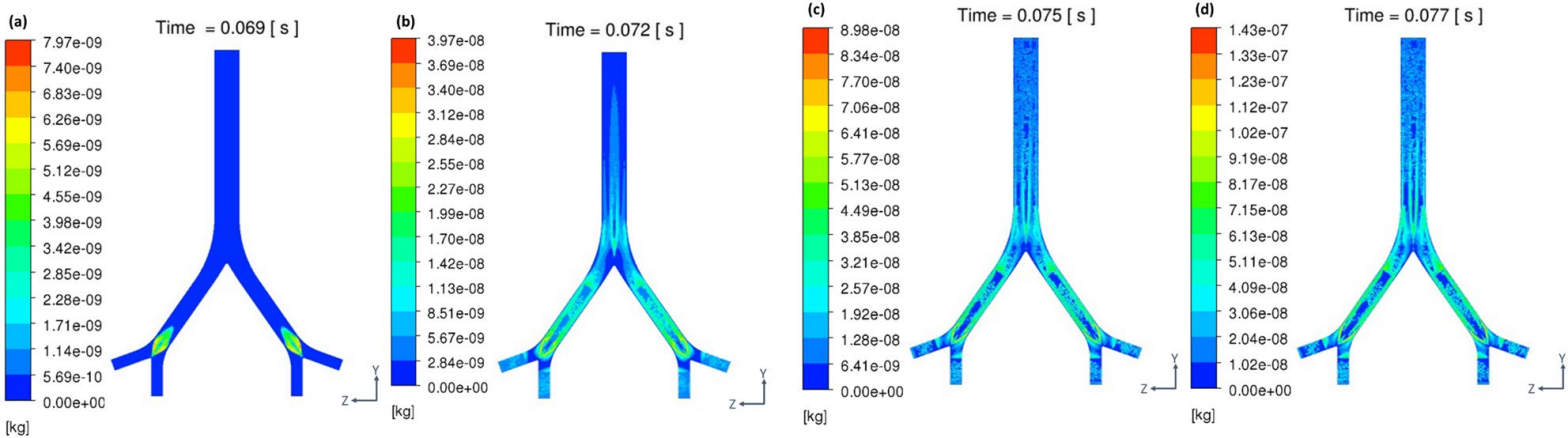
Stripped particle mass contour on the airway surface at PVT.

**Figure 6:**
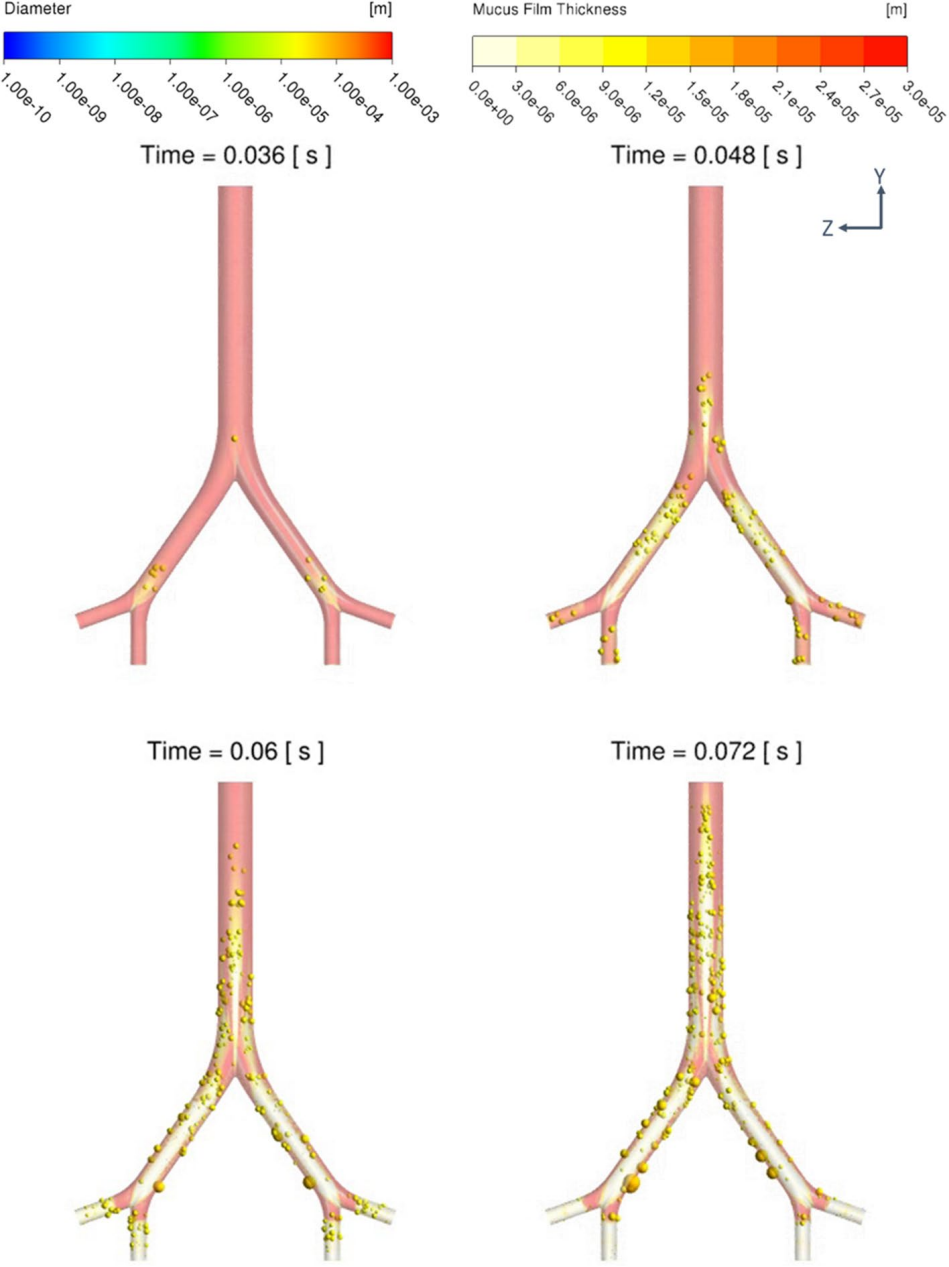
Generated droplets and thickness of the mucous film over time at CPFR = 4 L/s.

**Figure 7:**
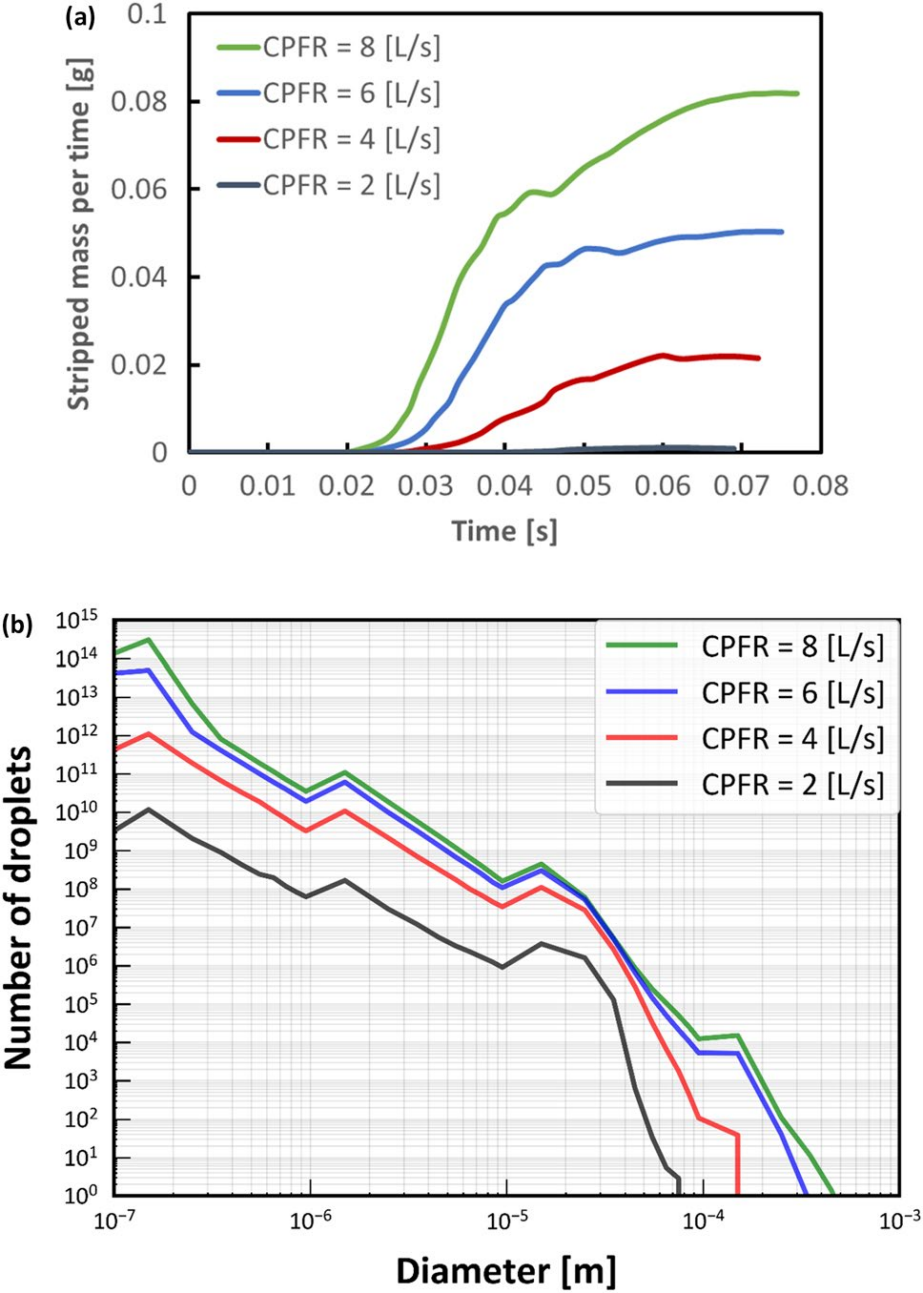
Particle stripping with different CPFRs. (**a**) Stripped particle mass of the mucous film for each time step. (**b**) Particle size distribution of droplets generated with different CPFRs.

Increasing the CFPR increased both the total mass and total number of stripped droplets: CFPR = 2, 4, 6, and 8 L/s resulted in total masses of 0.02, 0.61, 1.84, and 3.15 g, respectively, and total numbers of 0.06, 0.31, 1.18, and 1.41 × 10^20^, respectively. Figure 3 shows a contour plot of the velocity of the coughing airflow at PVT. The velocity was high at the center and low near the walls for G2 and G0. In contrast, the velocity was higher near the ventral and dorsal walls for G1. The maximum velocities at CPFR = 2, 4, 6, and 8 L/s were 12.8, 24.4, 36.1, and 47.9 m/s, respectively. Figure 4 shows a contour plot of the wall shear stress (WSS) generated by each cough at PVT. In the coronal plane, WSS decreased just before B2 and B1 and increased on the G2, G1, and G0 surfaces after the branches joined. WSS was lower on the lateral surface of the airway than on the front and back surfaces. Figure 5 shows a contour plot of the mass of droplets stripped by each cough at PVT. At CPFR = 2 L/s, droplet stripping only occurred on the G1 surface. At CPFR = 4 L/s or higher, droplet stripping occurred on other surfaces. The stripped droplet mass tended to be greater on the upper B2, G1, and B1 wall surfaces. Figure 6 shows the droplet generation and film thickness over time due to coughing at CPFR = 4 L/s. The left color bar indicates the particle size of the generated droplets, and the right color bar indicates the thickness of the airway wall mucosa. Droplet generation started from near the B2 wall and spread to G1, B1, and G0 over time. At PVT (bottom left in Fig. 6), there are almost no mucous liquid on B2. Figure 7a shows the change in the stripped droplet mass at each time step for the different CPFRs. Droplet stripping started at 0.034, 0.24, 0.021, and 0.019 s at CPFR = 2, 4, 6, and 8 L/s, respectively, which indicates that droplet stripping started earlier as CPFR was increased. Over time, the stripped droplet mass became saturated. For a given time step, the stripped droplet mass increased with CPFR, and the difference in peak values was about 70 times between CPFR = 2 and 8 L/s.

Figure 7b shows a histogram of the droplet size distribution at each CPFR. The number of droplets increased with a decreasing particle size, although some peaks were observed. The number of droplets increased with increasing CPFR. From CPFR = 2 to 4 L/s, the number of droplets increased uniformly for almost all particle sizes. From CPFR = 4 to 6 L/s, the number of droplets increased at particle sizes of 0.1–100 µm and 300–100 µm. At CPFR = 6 and 8 L/s, the number of droplets was particularly high at particle sizes of 0.1–100 µm and > 80 µm. The maximum droplet size of the droplets increased from 70 to 400 µm with an increasing CPFR.

### Influence of the mucous viscosity

At mucous viscosities of 1.00, 8.25, and 15.5 mPa·s, the stripped droplets had total masses of 0.61, 1.05, and 1.22 g, respectively, and total numbers of 3.14, 6.51, and 6.13 × 10^19^, respectively. The total mass of stripped droplets increased with increasing viscosity, but the number of droplets peaked at a viscosity of 8.25 mPa·s.

**Figure 8:**
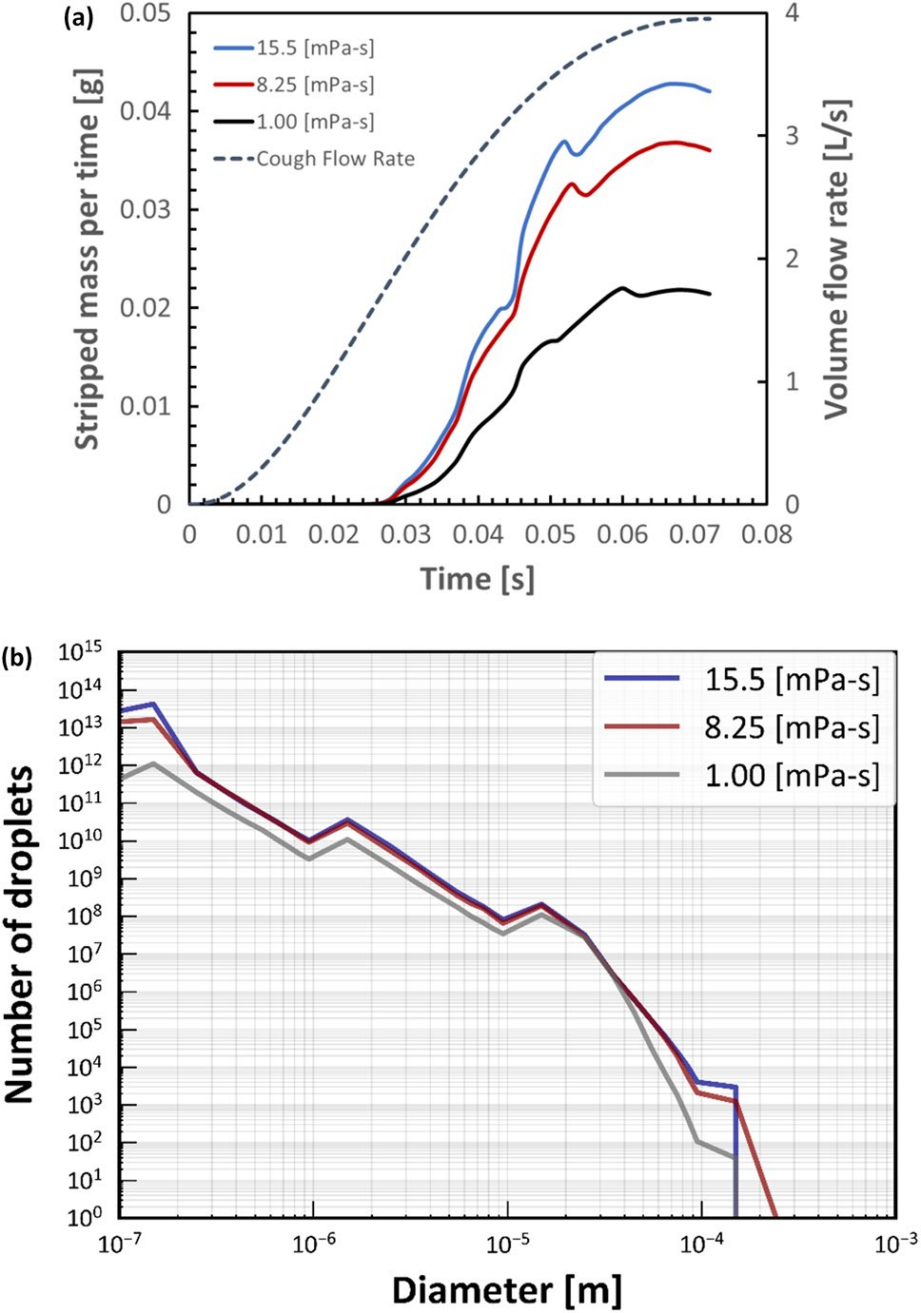
Particle stripping with different mucous viscosities. (**a**) Stripped particle mass of the film for each time step. (**b**) Particle size distribution of droplets generated.

Figure 8a shows the change in the stripped droplet mass for each time step at different mucous viscosities and CPFR fixed at 4 L/s. Particle stripping started at *t* = 0.024 s for all viscosities, and the curves of the stripped droplet mass peaked earlier than PVT. With increasing viscosity, the peak of the stripped droplet mass curve became more pronounced. The results showed that the stripped particle mass increased with increasing viscosity. Figure 8b shows a histogram of the particle size distribution of the generated droplets. The number of droplets generated increased with a decreasing particle size, although some peaks were observed. Increasing the viscosity from 1.00 to 8.25 mPa·s caused the number of droplets to increase for almost all particle sizes except at around 20 µm. Increasing the viscosity to 15.5 mPa·s increased the number of droplets at particle sizes of 0.1–0.2 µm and 100–200 µm. The maximum droplet size was similar at viscosities of 1.00 and 15.5 mPa·s, and the largest droplets were obtained at a viscosity of 8.25 mPa·s.

## Discussion

Three mechanisms of respiratory droplet generation associated with exhalation have been described in the literature: in the oral cavity, trachea–bronchi, and bronchioles^1^. However, almost no reports have quantified the number, mass, and particle size distribution of droplets generated by each mechanism. This study focused on reproducing the stripping of the airway wall mucosa due to airflow shear stress in the tracheal–bronchial region. The coupled DPM-EWF model was used to simulate droplet generation in the airway wall mucosa during coughing, and the effects of the mucous viscosity and cough flow rate on the number, mass, and particle size distribution of generated droplets were evaluated.

Various approaches have been used to measure the particle size distributions of respiratory droplets^8–26^. However, obtaining a comprehensive distribution experimentally is impossible because of differences in measurement methods and conditions. In addition, because particles emitted from the oral cavity are measured, studying the generation mechanisms separately is impossible. In this study, the coupled DPM-EWF model was applied to CFD analysis of droplet generation by the stripping of the mucous membrane in the airway by the coughing airflow, and the droplet generation and distribution were quantified.

The simulation results showed that a wide range of droplet sizes from 100 nm to 400 µm was generated. Because this method does not require a superfine mesh to resolve small droplets, it is applicable to large-scale geometries such as the entire airway. Furthermore, by applying this method to obtain the droplet distribution for various airway shapes, individual differences in droplet distributions can be considered for a more detailed simulation of droplet dispersion in the air.

Considering the algorithm of DPM-EWF coupling, WSS on airway wall is the dominant factor to control the droplet generation. And WSS is highly affected by the amount of airflow. Increasing the CFPR from 2 to 8 L/s increased the velocity of the internal airflow. In our ideal geometry, the maximum velocities were (Fig. 3) are close to the values reported by Kou et al.^43^ using patient specific airway geometry. Increasing the velocity also increased WSS (Fig. 4). Increasing WSS increased the stripped droplet mass of the droplets. A comparison between the stripped droplet mass distribution (Fig. 5) and WSS distribution (Fig. 4) indicates that more droplets tended to be generated in high-WSS regions.

Coupled DPM-EWF simulation can be applied for whole airway model and can exhibit the certain location of droplet generation. At CPFR = 2 L/s, droplet generation was only observed on the G1 surface. This may be because CSS was set to 1 Pa, and WSS was less than CSS for most airway walls other than that at G1. Lowering CSS should increase the droplet number because the region where CSS < WSS would increase. Increasing CPFR also increased the stripped droplet mass over time (Fig. 6), which suggests that a higher CPFR resulted in a larger area where WSS > CSS. The droplet stripping started earlier with increasing CPFR (Fig. 7a).

In our simulation, we introduce the initial thickness of mucous film, and film on airway wall does not replenish. Therefore, the maximum possible volume of stripped droplets is the mucus film thickness multiplied by the airway wall area. When CPFR = 8 L/s, the area where WSS > CSS becomes nearly 99% at PVT (Supplementary material). Compared to the increase in WSS with increasing CPFR, the increase in detachment with increasing CPFR is modest. This suggests that the amount of stripping may have already started to saturate at 8 L/s in the present simulation.

And as CPFR increases, maximum diameter of stripped droplet increases. Since the EWF is modeled based on the works of Lopez de Bertodano et al. and Mayer, the average particle size of the stripped droplets increases as the velocity of the airflow increases^33,34^.

In addition to the airflow, airway mucous condition also affects on droplet generation. The maximum number of stripped droplets and maximum droplet size were obtained at 8.25 mPa·s (Fig. 8b). The size distributions in different viscosities are almost similar comparing with that of different CPFRs. On the other hand, the droplets larger than 100 µm were generated only at 8.25 mPa·s. These large droplets may affect the total mass of generated droplets.

According to the studies of Lopez de Bertodano et al. and Mayer, liquid film viscosity will have a positive correlation^33,34^. However, total mass of generated droplet has a linear correlation, whereas the total number and the maximum diameter do not have a linear correlation. In this study, CPFR and viscosity were used as parameters. However, there must be other parameters that affect the results, such as model geometry. More detailed parametric studies are needed in the future to investigate the mechanism of droplet generation and response to parameters in EWF modeling of cough.

To partially corroborate the results using EWF, we described the comparison between our EWF model and VOF method. Pairetti et al. used the VOF method to reproduce droplet generation with a uniform profile of a 30 m/s airflow on a flat liquid film, which resulted in a maximum droplet size of about 400 µm^30^. In the present study, the maximum droplet diameter had a range of 70–400 µm depending on the CPFRs considered. The maximum flow velocity was 47.9 m/s at CPFR = 8 L/s, which resulted in a maximum droplet size at about 400 µm and agrees with Pairetti et al.’s results. On the other hand, the minimum diameter is not comparable since Pairetti et al.^30^ tracked a minimum droplet size of 100 µm. The VOF requires an extremely fine mesh to track droplet behavior at the microscale or nanoscale because the droplet size depends on mesh resolution. Therefore, for airway tree geometries with complex 3D structures, simulating the generation of droplets with a wide particle size distribution requires a fully resolved mesh and is very computationally expensive. In the present study, droplets were generated with a minimum droplet size of 0.1 nm, but only droplets larger than 0.1 µm were counted because coronaviruses are generally around that size.

The droplet size distribution in the present study showed that smaller droplets were generated in larger numbers. Because only shear-induced droplets in the lower airway were considered and the droplets generated were counted rather than the droplets released from oral cavity, the obtained droplet size distributions were significantly different from those obtained experimentally in previous studies^8–26^. For many of these studies, the droplet size distributions peaked at the microscale. Guo et al.^44^ simulated the droplet deposition during expiration by using the geometry of the entire respiratory system, including the vocal cords and oral cavity. Their results showed that the arrival rate of droplets in the oral cavity was affected by the expiratory flow rate and droplet size, and very small droplets were deposited in the pharyngeal region. Therefore, modeling from the pharynx to the oral cavity may significantly reduce the number of droplets that reach the oral cavity, especially small droplets.

The presence or absence of relative humidity outside the oral cavity should greatly affect the results of both numerical simulations and experimental measurements. The relative humidity in the human respiratory tract is usually 100% owing to its high humidity compared with the surrounding environment. Therefore, when droplets are released from the respiratory tract into the atmosphere, the water in the droplets evaporates, which reduces their diameter. Because the evaporation rate is generally faster with a smaller droplet size^45^, in the actual environment, nanoscale droplets may evaporate completely before reaching the measurement device or become smaller than the measurement resolution. Thus, they are unlikely to be captured by the particle size distribution measured outside the oral cavity.

In this study, Weibel’s model was used to represent the ideal airway shape, in which the airway cross-section is a circle and symmetric branches form with smooth airway walls. However, the actual human body has a more complex and asymmetric airway shape. The wall surface was simplified to be a rigid wall that does not move. When a fast airflow is expelled such as by coughing, the cross-sections of the trachea and bronchi may be deformed without the support of cartilage. By creating an airway model considering left–right asymmetry and a patient-specific model of expiration constructed from dynamic computed tomography, the methods used in this study can obtain droplet distributions for complex airway shapes.

Although various protein components are dissolved in body fluids, a single-phase liquid film comprising a Newtonian fluid with uniform viscosity was applied to the airway walls in this study. Because the fluid film moves during coughing and results in a nonuniform shear rate, applying a non-Newtonian model to the mucous membrane viscosity may reproduce more complex fluid film behavior by showing localized changes in viscosity.

## Conclusion

The aim of this study was to identify quantitative features related to droplet generation and to simulate droplet generation in the airway wall mucosa during coughing. CFD analysis was coupled with DPM-EWF to reproduce droplet generation from the airway mucosa due to WSS. Droplet stripping was observed especially in high-WSS areas resulting from the confluence of bronchioles. A smaller particle size increased the number of droplets generated. Increasing CPFR resulted in increasing WSS, and consequently higher WSS increased the number, mass, and maximum particle size of the droplets. Increasing the mucous viscosity also increased the mass of the generated droplets. The coupled DPM-EWF model can be applied to complex geometries, such as patient-specific airways, with less computational cost. The methods used in this study can be used to quantify the particle size distribution and maximum particle diameter of generated droplets under various conditions.

## Supplementary Information

Supplementary Information.

## Data Availability

The raw and processed data are available from the corresponding authors on reasonable request.

## References

[1] Dhand R, Li J (2020). Coughs and sneezes: Their role in transmission of respiratory viral infections, including SARS-CoV-2. Am. J. Respir. Crit. Care Med..

[2] Zhu SW, Kato S, Yang JH (2006). Study on transport characteristics of saliva droplets produced by coughing in a calm indoor environment. Build. Environ..

[3] Chen C, Zhao B (2010). Some questions on dispersion of human exhaled droplets in ventilation room: Answers from numerical investigation. Indoor Air.

[4] Yan YH, Li XD, Tu JY (2019). Thermal effect of human body on cough droplets evaporation and dispersion in an enclosed space. Build. Environ..

[5] Feng Y, Marchal T, Sperry T, Yi H (2020). Influence of wind and relative humidity on the social distancing effectiveness to prevent COVID-19 airborne transmission: A numerical study. J. Aerosol Sci..

[6] Fontes D, Reyes J, Ahmed K, Kinzel M (2020). A study of fluid dynamics and human physiology factors driving droplet dispersion from a human sneeze. Phys. Fluids.

[7] Busco G, Yang SR, Seo J, Hassan YA (2020). Sneezing and asymptomatic virus transmission. Phys. Fluids.

[8] Gerone PJ, Couch RB, Keefer GV, Douglas RG, Derrenbacher EB, Knight V (1966). Assessment of experimental and natural viral aerosols. Bacteriol. Rev..

[9] Duguid JP (1946). The size and the duration of air-carriage of respiratory droplets and droplet-nuclei. J. Hyg..

[10] Jennison MW (1942). Atomizing of mouth and nose secretions into the air as revealed by high speed photography. Aerobiology.

[11] Fennelly KP, Martyny JW, Fulton KE, Orme IM, Cave DM, Heifets LB (2004). Cough-generated aerosols of mycobacterium tuberculosis: A new method to study infectiousness. Am. J. Respir. Crit. Care.

[12] Edwards DA, Man JC, Brand P, Katstra JP, Sommerer K, Stone HA, Nardell E, Scheuch G (2004). Inhaling to mitigate exhaled bioaerosols. Proc. Natl. Acad. Sci. U. S. A..

[13] Yang SH, Lee GWM, Chen CM, Wu CC, Yu KP (2007). The size and concentration of droplets generated by coughing in human subjects. J. Aerosol Med..

[14] Fabian P, McDevitt JJ, DeHaan WH, Fung ROP, Cowling BJ, Chan KH, Leung GM, Milton DK (2008). Influenza virus in human exhaled breath: An observational study. PLoS ONE.

[15] Chao CYH, Wan MP, Morawska L, Johnson GR, Ristovski ZD, Hargreaves M, Mengersen K, Corbett S, Li Y, Xie X, Katoshevski D (2009). Characterization of expiration air jets and droplet size distributions immediately at the mouth opening. J. Aerosol Sci..

[16] Xie XJ, Li YG, Sun HQ, Liu L (2009). Exhaled droplets due to talking and coughing. J. R. Soc. Interface.

[17] Morawska L, Johnson GR, Ristovski ZD, Hargreaves M, Mengersen K, Corbett S, Chao CYH, Li Y, Katoshevski D (2009). Size distribution and sites of origin of droplets expelled from the human respiratory tract during expiratory activities. J. Aerosol. Sci..

[18] Haslbeck K, Schwarz K, Hohlfeld JM, Seume JR, Koch W (2010). Submicron droplet formation in the human lung. J. Aerosol Sci..

[19] Holmgren H, Ljungstrom E, Almstrand AC, Bake B, Olin AC (2010). Size distribution of exhaled particles in the range from 0.01 to 2.0 μm. J. Aerosol Sci..

[20] Han ZY, Weng WG, Huang QY (2013). Characterizations of particle size distribution of the droplets exhaled by sneeze. J. R. Soc. Interface.

[21] Buckland FE, Tyrrell DAJ (1964). Experiments on the spread of colds: 1. Laboratory studies on the dispersal of nasal secretion. J. Hyg..

[22] Hersen G, Moularat S, Robine E, Gehin E, Corbet S, Vabret A, Freymuth F (2008). Impact of health on particle size of exhaled respiratory aerosols: Case-control study. Clean.

[23] Johnson GR, Morawska L (2009). The mechanism of breath aerosol formation. J. Aerosol Med. Pulm. Drug Deliv..

[24] Almstrand AC, Bake B, Ljungstrom E, Larsson P, Bredberg A, Mirgorodskaya E, Olin AC (2010). Effect of airway opening on production of exhaled particles. J. Appl. Physiol..

[25] Lindsley WG, Pearce TA, Hudnall JB, Davis KA, Davis SM, Fisher MA, Khakoo R, Palmer JE, Clark KE, Celik I, Coffey CC, Blachere FM, Beezhold DH (2012). Quantity and size distribution of cough-generated aerosol particles produced by influenza patients during and after illness. J. Occup. Environ. Hyg..

[26] Milton DK, Fabian MP, Cowling BJ, Grantham ML, McDevitt JJ (2013). Influenza virus aerosols in human exhaled breath: Particle size, culturability, and effect of surgical masks. PLoS Pathog..

[27] Mittal R, Ni R, Seo JH (2020). The flow physics of COVID-19. J. Fluid Mech..

[28] Vadivukkarasan M, Dhivyaraja K, Panchagnula MV (2020). Breakup morphology of expelled respiratory liquid: From the perspective of hydrodynamic instabilities. Phys. Fluids.

[29] Grotberg JB (2001). Respiratory fluid mechanics and transport processes. Annu. Rev. Biomed. Eng..

[30] Pairetti C, Villiers R, Zaleski S (2021). On shear layer atomization within closed channels: Numerical simulations of a cough-replicating experiment. Comput. Fluids.

[31] Tao Y, Huai X, Guo Z, Yin R (2009). Numerical simulation of spray performance based on the Euler–Lagrange approach. J. Therm. Sci..

[32] Ashmore J, Hosoi AE, Stone HA (2003). The effect of surface tension on rimming flows in a partially filled rotating cylinder. J. Fluid Mech..

[33] Lopez de Bertodano MA, Jan C-S, Beus SG (1997). Annular flow entrainment rate experiment in a small vertical pipe. Nucl. Eng. Des..

[34] Mayer S (1961). Theory of liquid atomization in high velocity gas streams. ARS J..

[35] Friedrich MA, Lan H, Wegener JL, Drallmeier JA, Armaly BF (2008). A separation criterion with experimental validation for shear-driven films in separated flows. J. Fluids Eng..

[36] Paz C, Suarez E, Parga O, Vence J (2017). Glottis effects on the cough clearance process simulated with a CFD dynamic mesh and Eulerian wall film model. Comput. Methods Biomech. Biomed. Eng..

[37] Paz C, Suarez E, Vence J (2017). CFD transient simulation of the cough clearance process using an Eulerian wall film model. Comput. Methods Biomech. Biomed. Eng..

[38] Ren S, Shi Y, Cai M, Zhao H, Zhang Z, Zhang XD (2018). Ansys-matlab co-simulation of mucus flow distribution and clearance effectiveness of a new simulated cough device. Int. J. Numer. Methods Biomed. Eng..

[39] Ren S, Li W, Wang L, Shi Y, Cai ML, Hao LM, Luo ZH, Niu JL, Xu WQ, Luo ZJ (2020). Numerical analysis of airway mucus clearance effectiveness using assisted coughing techniques. Sci. Rep..

[40] Weibel ER (1979). Morphometry of the human-lung: The state of the art after 2 decades. Bull. Eur. Physiopathol. Res..

[41] Rajendran RR, Banerjee A (2019). Mucus transport and distribution by steady expiration in an idealized airway geometry. Med. Eng. Phys..

[42] Gupta JK, Lin CH, Chen Q (2009). Flow dynamics and characterization of a cough. Indoor Air.

[43] Kou GY, Li XH, Wang Y, Lin MY, Zeng YP, Yang XP, Yang YY, Gan ZM (2018). CFD simulation of airflow dynamics during cough based on CT-scanned respiratory airway geometries. Symmetry.

[44] Guo Y, Wei JJ, Ou CY, Liu L, Sadrizadeh S, Jin T, Tang LL, Zhang YP, Li YG (2020). Deposition of droplets from the trachea or bronchus in the respiratory tract during exhalation: A steady-state numerical investigation. Aerosol Sci. Technol..

[45] Xie X, Li Y, Chwang ATY, Ho PL, Seto WH (2007). How far droplets can move in indoor environments—Revisiting the wells evaporation-falling curve. Indoor Air.

