# Supplementary material for: Coupled discrete phase model and Eulerian wall film model for numerical simulation of respiratory droplet generation during coughing
Source: Sci Rep. 2022 Sep 1;12:14849. doi: 10.1038/s41598-022-18788-3 (PMC9434508; doi:10.1038/s41598-022-18788-3)

Supplementary material

The figure shows the distribution of WSS and stripped mass at PVT in different CPFRs. In this simulation, the thickness of the mucus film is introduced as the initial value; the film on the airway wall is not replenished. Therefore, the thickness of the droplet film multiplied by the area of the airway wall may be the maximum threshold value. Considering CSS, one of the parameters controlling droplet detachment, we found that at 8 L/s CPFR, the area ratio of CSS<WSS is almost 99% at PVT; the increase in detachment with increasing CPFR is slower than the increase in WSS with increasing CPFR. This suggests that the detachment volume may have already begun to saturate at 8 L/s.


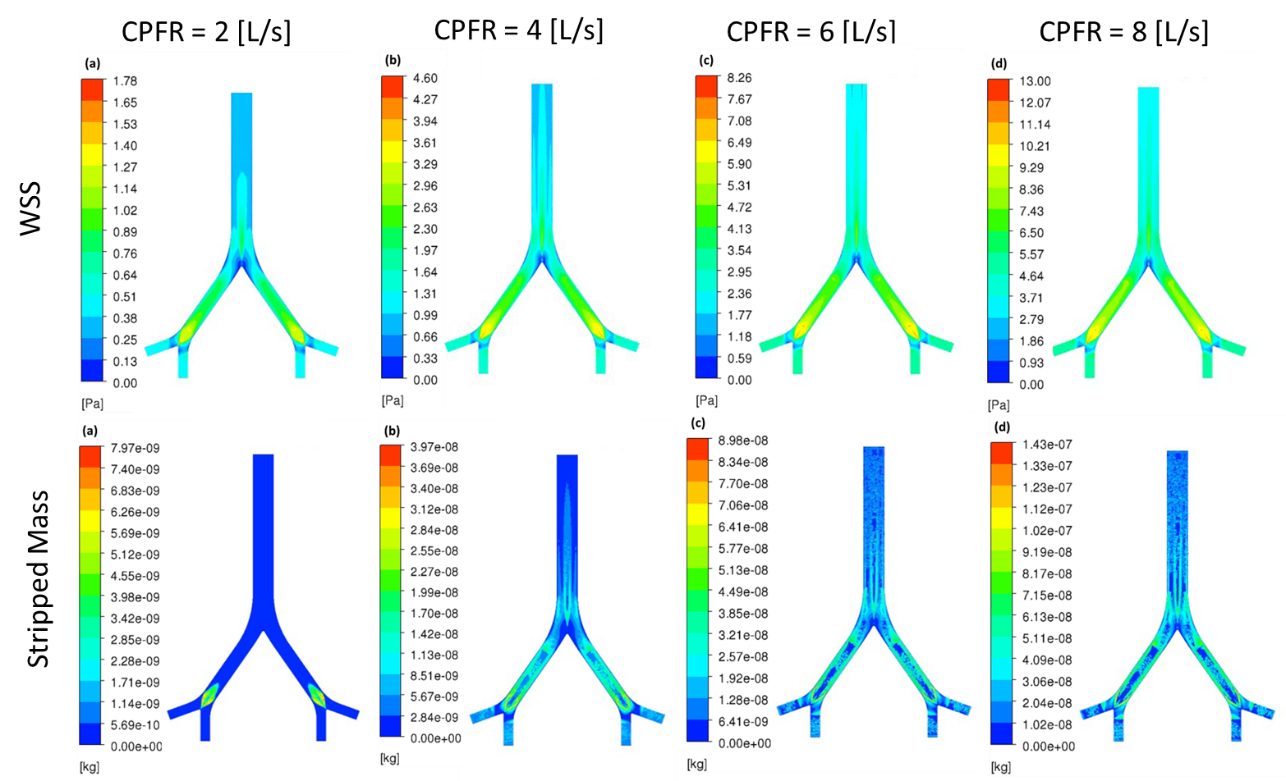

Supplement: Supplementary file 1 — Supplementary Information. [file 41598_2022_18788_MOESM1_ESM.docx]
